# Social relationships and physician utilization among older adults—A systematic review

**DOI:** 10.1371/journal.pone.0185672

**Published:** 2017-09-28

**Authors:** Daniel Bremer, Laura Inhestern, Olaf von dem Knesebeck

**Affiliations:** 1 University Medical Center Hamburg-Eppendorf, Department of Medical Psychology, Hamburg, Germany; 2 University Medical Center Hamburg-Eppendorf, Center for Health Care Research, Hamburg, Germany; 3 University Medical Center Hamburg-Eppendorf, Department of Medical Sociology, Hamburg, Germany; Universita degli Studi di Firenze, ITALY

## Abstract

**Background:**

In older age health needs and demand for health services utilization increase. Individual’s social relationships can play a decisive role regarding the utilization of outpatient health care services. This systematic review examines the associations of structural and functional dimensions of social relationships with outpatient health services use of older adults.

**Methods:**

The databases PubMed, CINAHL, SocINDEX, PsycINFO, International Bibliography of the Social Sciences (IBSS), Sociological Abstracts, and Applied Social Sciences Index and Abstracts (ASSIA) were searched in February 2016. The methodological and reporting quality of the articles was assessed and the results were synthesized descriptively and systematically.

**Results:**

Out of 1.392 hits, 36 articles (35 studies) were included in the systematic review. The methodological and reporting quality of the included articles was reasonable. Various structural and functional characteristics of social relationships were associated with the use (yes/no) and the frequency of using outpatient care among older adults. The majority of the associations between structural dimensions of social relationships and the use of physicians were positive and moderate in strength. The associations between functional dimensions of social relationships and the probability of using physician services were inconsistent and varied in strength. For the most part, social relationship variables assigned to the structural dimension were positively and weakly to moderately associated with the frequency of physician visits. Functional aspects of social relationships also tended to have positive associations with the frequency of physician utilization. The associations were weak to moderate in strength.

**Conclusions:**

Measuring social relationships and their influence on health services use is a challenging methodological endeavor indicated by the inconclusive results. The results suggest that the outpatient care utilization behavior of older individuals being structurally and functionally integrated in social relationships is different to older adults being socially isolated or having no social support. All in all, the current status of quantitative data was insufficient. Future health services research should accentuate social ties in more detail, especially according to quality aspects of social relationships.

## Background

Rapidly ageing populations generate increasing health needs and chronic conditions in Western industrial countries associated with a rising demand for health services [1]. Compared with younger cohorts, individuals within their fifties or older show more chronic illnesses and increased rates of health care use [2]. Utilization of health services is influenced by a variety of factors, e.g. predisposing, enabling and need characteristics [3]. These are constantly changing over the life course. Due to chronic conditions and physical limitations in older age need factors can expand, while levels of autonomy, mobility and social participation decrease [4]. The use of health services is embedded into a complex structure of social networks and interactions. Social relationships can be an enabling determinant of whether or not elderly individuals do consult health care services [5, 6]. Consequently, the question if social relationships buffer or foster the use of medical care has been raised within health services research [7–13]. Social relationships may be an opportunity to enable the use of health services, especially for vulnerable groups. Moreover, they can be used to support or substitute formal health services, and by that, release restricted resources in health care systems.

Following Berkman, Glass [14] social ties have an effect on individuals by providing social support, social influence, social engagement and attachment, and accessing resources and material goods. Beyond that, international studies have shown that social relationships have a substantial impact on morbidity and mortality [15–18]. In general, social relationships can be divided into structural and functional elements [15]. The degree of social network integration, a more quantitative measure, represents the structural dimension of social relationships (e.g. living arrangements, social network size, and frequency of social participation). The functional perspective is captured by received and perceived social support, and includes aspects of financial, instrumental, informational or emotional support. Through preventive care-seeking, acquisition of knowledge about potential treatments, and post-treatment recovery and rehabilitation, health service utilization behavior can be considered a health-protective action influenced by structural and functional aspects of social relationships [7]. A principal element in most health care systems is presented by outpatient health services, including primary and secondary care. Although, the first contact to health care is realized routinely through primary care services (e.g. in the US and UK), the close linkage to specialists and ambulatory health services is a ubiquitous characteristic within health care systems. By taking into account the political and scientific debate of shifting health care services from inpatient to outpatient settings, outpatient health services will be of growing importance in the future.

To date, no systematic review on this topic has been conducted. Therefore, the first aim of this systematic review was to provide an overview of studies dealing with outpatient care utilization among older adults associated with various dimensions of social relationships. The second aim was to evaluate magnitude and consistency of the associations between social ties and health services use.

## Method

A systematic review on studies dealing with social relationships and the utilization of outpatient care physicians among older adults was conducted. The performance of this review was based on the PRISMA checklist [19] and a study protocol including all preliminary specifications published on PROSPERO, registration number CRD42016036004 (S1 File, S1 Table).

### Search strategy and inclusion criteria

After developing the research question and performing a pilot run of literature search, seven databases were used (February 11^th^ 2016). The databases PubMed, CINAHL, SocINDEX, PsycINFO, International Bibliography of the Social Sciences, Sociological Abstracts, and Applied Social Sciences Index and Abstracts were searched for the keywords and various synonyms “social relationships”, “utilization”, “outpatient care” and “aged” in title and abstract (S1 Text). MeSH-terms and limiters were adapted to each electronic database. In addition, references of relevant articles were searched for further matching studies.

At first, one reviewer (DB) screened the titles and abstracts of all articles identified by electronic and reference search. In a second step, two independent reviewers (DB and LI) applied a predefined set of inclusion criteria on all relevant articles by performing a full text screening. In case of disagreement between the reviewers, a third investigator (OK) was consulted and the study was discussed until consensus was accomplished.

Within the full text screening, articles had to pass five predefined inclusion criteria. Firstly, records were controlled for the criterion “peer-reviewed journal articles in German or English”. Peer-reviewed journal articles represent good scientific practice to secure quality, to foster objectivity and to provide transparency. Due to language skills and a reasonable use of resources of the reviewers, German and English articles were screened. Secondly, records were checked for three different study designs: quantitative observational 1) cross-sectional, 2) case-control and 3) cohort studies. Thirdly, full texts were inspected for the criterion “community-dwelling or noninstitutionalized individuals fifty years and older”. The rationale behind this population was to extract a reference group still active on the labor market, and to expand the number of potentially relevant studies. Compared with younger cohorts, individuals within their fifties or older show more chronic illnesses and increased rates of health care use [2]. The fourth inclusion criterion was the accounting for utilization or frequency of use of outpatient care services as the dependent or outcome variable. These measures of use are solidly established in health services research and increase the chance of comparability. Finally, studies had to include and analyze social relationship variables. To gather information on the full spectrum of social relationships including structural and functional aspects this broad term was implemented.

### Data extraction and quality assessment

The data was extracted using a standardized form including information about the author, year, country, research design, study year (follow-up if applicable), sample size, response rate, age, gender, outcome, social relationship variables, and confounders in the fully adjusted model.

The quality assessment, including the methodological and reporting quality, was based on a checklist following the Newcastle-Ottawa-Scale [20] and its adaptation of Herzog, Alvarez-Pasquin [21]. The checklist included the three sections “selection”, “comparability and confounders” and “outcome”. It consisted of ten (cohort studies) respectively eight items (cross-sectional studies) which could be answered by “yes”, “no” or “unclear”. Instead of reporting a sum score, a global rating was preferred [22]. The quality of cross-sectional studies which met three or less criteria were ranked as “low”, four or five as “medium” and six or more as “high”. Cohort studies with four or less fulfilled criteria were rated as “low” quality, five to seven as “medium” and eight or more as “high”.

### Analysis strategy

The results were descriptively and systematically synthesized. All associations between social relationships and utilization of physicians were extracted and categorized. Each social relationship variable was assigned to a social relationship category and dimension. For a better overview, closely related indicators were aggregated within categories (e.g., marital status or social support). Moreover, social relationship variables were classified as “structural” or “functional” [15]. The functional dimension was split into “received support” and “provided support”. To answer our two research questions, we looked comprehensively at all associations between social relationships and physician use. For the sake of clarity and presentation, we focused on the statistically significant associations in our following tables (p<0.05). Due to the heterogeneity of the included studies a meta-analysis was not performed. Instead, we decided to complement our descriptive analysis by assessing the quality of the studies and by presenting a full description of the relevant quantitative data to maximize transparency and to enable rating the certainty of the results [23]. Since use (yes/no) and frequency of practitioner visits show a distinct level of information and have different meanings, the results are reported separately.

## Results

### Literature search

**Fig 1:**
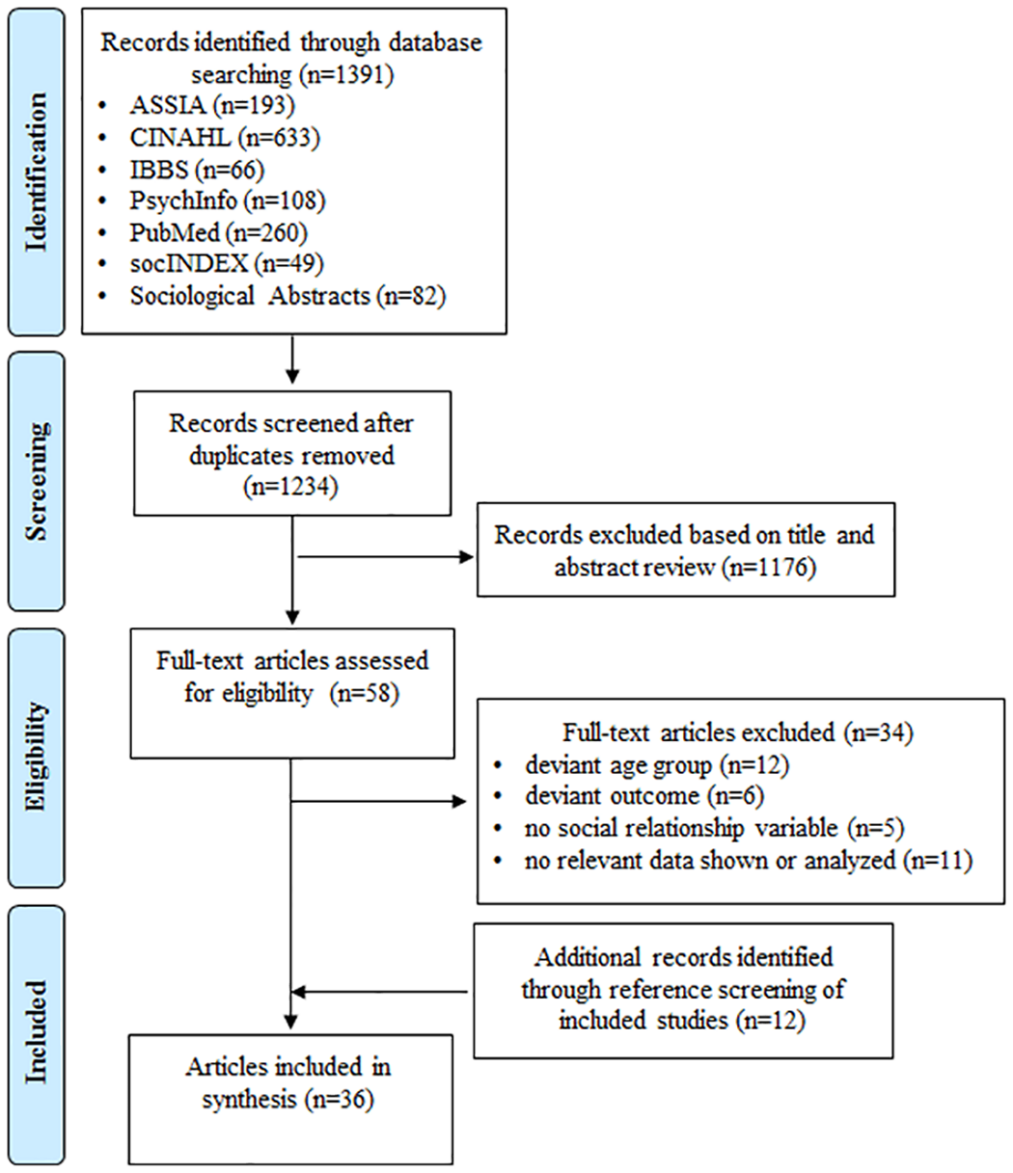
Flow chart of systematic literature search.

A total of 1,392 publications were identified through database search. After removing 158 duplications, 1,234 articles remained for title and abstract screening (Fig 1). 1,176 publications were excluded based on title and abstract screening. Fifty-eight full-text articles were assessed for eligibility (S3 Table). Thirty-four were eliminated due to various reasons (deviant age group, deviant outcome, no social variable, none relevant data shown or analyzed). Twelve records were identified through reference search of included articles. In the full text screening inter-rater agreement on study inclusion was 88%. In the end, thirty-six publications based on thirty-five studies were included in the review and the synthesis. Though two articles [8, 24] were based on the same study, their methodological and reporting quality was evaluated separately and their results were analyzed independently due to differing samples and data sets.

### Overview of included records

The articles were published between 1981 and 2015 (Table 1). More than half of the records were from the USA (20; 55.6%). Eight articles were from Europe (22.2%), five from Asia (13.9%), two from Canada (5.6%) and one from Australia (2.8%). The sample sizes ranged from N = 40 to N = 824,952 and mean age ranged from 63 to 81 years. Two studies focused on women only [10, 25], the others had quota of women of 45% to 66%. Twenty-two studies were cross-sectional and fourteen were prospective cohort studies. Twenty-three studies analyzed the frequency of physician visits (ordinal, metric or count variables). Nine studies researched the use of physicians (yes vs. no). Four articles [12, 26–28] reported both outcomes and therefore, they were listed in the “use” and “frequency” section. The period of outpatient care use ranged from fifteen days to two years. More than half of the articles focused explicitly on GP visits [26, 29–34], primary care [35–38], ambulatory services [8, 24, 39–42], and outpatient physician visits [7, 43, 44]. The other records used more implicit terms like “physicians” [45], “doctors” [46] and “consultations” [34] in contrast to inpatient health care services (e.g. hospital days, hospital nights).

**Table 1:** Overview of characteristics of included studies.

Author(s), year, country	Research design (specific population)	Study year (follow-up)	Sample size	Response rate in %	Age	Female in %	Covariates in fully adjusted model
Arling, 1985, USA [8]	cross-sectional study	1979	2,051	87	60–64: 29% 65–74: 47% 75–84: 19% 85+: 5%	59	medical conditions, ADL impairment, psychosomatic symptoms, emotional symptoms, economic deprivation, insurance coverage, medical care source, age, education, sex, race
Branch et al., 1981, USA [42]	cross-sectional study	1974	1,625	79	73.2 (mean)	60	age, gender, race, education, income, occupation, health insurance, regular physician, transportation problems, perceived health status, activities of daily living, physical activity performance, ability to climb stairs, ability to walk half a mile, health problem
Cafferata, 1987, USA [48]	cross-sectional study	1977	4,560	n.a.	73.5 (mean)	60	race, education, chronic condition, health insurance, density of physicians, health, worry, physicians usual source of care, bed-disability days
Coulton & Frost, 1982, USA [41]	cohort study	1975 (1976)	1,834 (1,519)	n.a.	74.2 (mean)	65	perceived service need, level of impairment, income, education, insurance, case management, gender, age, race, psychic stress
Counte & Glandon, 1991, USA [28]	cohort study (health maintenance organization members and fee-for-service clients)	1986 (+6 months)	402	74 & 44 (87 & 85)	72.5 (mean)	63	health status, life stress, insurance, SES, gender
Crespo-Cebada & Urbanos-Garrido, 2012, Spain [26]	cross-sectional study	2006/07	1,860	n.a.	n.a.	n.a.	age, gender, longillness, symptoms, chronic diseases, limitations, depression, orientation, health, physical activity, education, job status, insurance, income, homecare
Dalsgaard et al., 2012, Denmark [29]	cohort study (diabetes cohort)	2003 (2009)	824,952	n.a.	55–64: 33.4% 65–79: 40.6%	45	sex, age, education, occupation, income
Eve, 1988, USA [25]	cohort study (older women cohort)	1969 (1979)	3,013 (1,849)	62.9 (61.4)	70.4 (mean)	100	age, education, race, head of household, retirement status, income, satisfied with way of living, able to get along on income, health insurance, metropolitan area, handicapped/disabled, health compared to others, previous use of health services
Ezeamama et al., 2015, USA [46]	cross-sectional study	2010/11	4,562	80	50–55: 22.36% 56–60: 25.71% 61–65: 24.38% 66–70: 27.55%	57.8	history of loss, age, sex, education, smoking, BMI, physical activity level, US-born, fall, trouble sleeping, race, cumulative lifetime adversity, global mastery, domain-specific mastery, importance of religion, comorbidities, retirement status
Foreman et al., 1998, China [9]	cross-sectional study	1998	350	n.a.	71.6 (mean)	51.4	gender, age, education, alcohol
Fritel et al., 2014, France [10]	cohort study (urinary incontinence cohort, women only)	2000 (2008)	2,640 (2,273)	n.a. (86)	63 (mean)	100	age, parity, urinary incontinence (UI) severity at baseline, UI type, quality of life, consultation with GP in the last 12 months, neurologic disease, hypertension or cardiovascular disease
Gobbens & van Assen, 2012, The Netherlands [30]	cohort study	2008 (2009, 2010)	245 (179, 141)	53 (73, 58)	80.3 (mean)	54.7	sex, age, lifestyle, multimorbidity, physical frailty, psychological frailty, social frailty, BMI, activities, fatigue, mobility, balance, hand grip strength, depression, anxiety, coping, mental state
Goldsteen et al., 1992, USA [43]	cohort study	1986 (+6 months)	402 (346)	59.6 (86.1)	72.5 (mean)	63	age, sex, race, education, religion, health locus control, physician visits t0, desirable life events, activities, health problems, HMO, Eldercare
Hand et al., 2014, Canada [35]	cross-sectional study (frequent health services user)	n.a.	40	44.9	81.3 (mean)	55	health status
Harris et al., 2004, UK [34]	cohort study	2000 (2001)	1,565	75 (92)	65–69: 24% 70–74: 25% 75–79: 22% 80–84: 15% 85+: 14%	62	age, sex, practice, general health, disease score, anxiety score
Jordan et al., 2006, UK [31]	cohort study (knee pain cohort)	2000 (-/+18 months)	1,797	77 (100)	n.a.	n.a.	knee-related factors, general health, sex, age, education
Korten & Jacomb, 1998, Australia [32]	cohort study	1990/91 (1994)	897 (624)	65 (85)	76.4 (mean)	n.a.	number of current illnesses, level of pain
Krause, 1988, USA [11]	cohort study (stress cohort)	1984 (+18 months)	351 (265)	n.a. (75.5)	n.a.	n.a.	age, sex, education, physical health status
Levkoff et al., 1987, USA [47]	cohort study (middle-aged and aged cohorts)	n.a.	152	88 (n.a.)	n.a.	n.a.	gender, education, has preventive outlook, thinks appropriate to talk to doctor about personal problems
Li & Chi, 2011, China [12]	cross-sectional study	2000	20,255	98.6	69.1 (mean)	47	age, gender, education, place of residence, income, health insurance, convenience of visiting a physician, self-rated health, functional health
Liao et al., 2012, Taiwan [44]	cohort study (introduction of national health insurance cohort)	1993 (1996)	2,230 (1,504)	90 (67.4)	69.7–71.1 (means)	35–62	age, gender, education, employment status, lifestyle behaviors, ethnicity, health/chronic conditions
Miltiades & Wu, 2008, China & USA [49]	cross-sectional study (chinese immigrants)	2000–03	597	88,5 & 91	69.7–71.8 (means)	62.1 & 59.3	education, traditional chinese medicine, self-rated health, depression (CES-D), chronic conditions, income, insurance, residence
Park, 2012, South Korea [38]	cross-sectional study	2003	6,591	94.1	n.a.	n.a.	age, gender, education, religion, self-perceived health status, cognitive condition, income, health insurance
Pourat et al., 2000, USA [39]	cross-sectional study (korean immigrants)	1993	424	n.a.	73–75 (means)	60 & 65	demographics, health, functioning, income, insurance, perceptions of health/other beliefs
Rennemark et al., 2009, Sweden [37]	cross-sectional study (frequent health services user)	2001–03	643	72.8	66 (mean)	54.2	age, gender, functional ability, comorbidity, education, sense of coherence, internal locus of control
Ryvicker et al., 2012, USA [36]	cross-sectional study	2008	1,260	76.7	75.4 (mean)	65	supply quartile, neighborhood safety, use public transit, age, female, nonwhite, non-English speaking, education, health insurance, usual source of care, number of chronic conditions, number of ADL/IADL needs
Schafer, 2013, USA [7]	cross-sectional study	2005/06	3,005	75.5 & 84	69.3 (mean)	52	sex, age, education, ethnicity, self-rated health, disease, regular place for health care, health insurance, alternative medicine
Schmitz et al., 1997, USA [6]	cohort study	n.a.	226	55 (n.a.)	n.a.	n.a.	daily hassles, age, depression, physical health, number of health problems
Stoller, 1982, USA [27]	cross-sectional study	1979	753	71	73.2 (mean)	57	symptoms, cancer effects, heart disease effects, stroke effects, worry about health, health interferes, ill in bed, health insurance, finances tight, care at MD's office, availability inconvenient, MD/population ratio, health attitudes, education, rural/urban, age, sex
Strain, 1990, Canada [13]	cross-sectional study	1985	705	75	71 (mean)	59	perceived health, number of chronic conditions, functional disability, health beliefs, age, gender, education, occupation, ethnic identity, religion, income
Suominen-Taipale et al., 2004, Norway & Finland [33]	cross-sectional study	1995–97	9,202	71 & 86	65–69: 49–57% 70–74: 43–51%	53 & 33	sex, age, self-rated health, education, region
Wan & Arling, 1983, USA [24]	cross-sectional study (functionally impaired, subsample Arling 1985)	1979	772	n.a.	72.6 (mean)	62.2	age, sex, race, residential background, occupation, education, income, health insurance, regular physician, perceived service needs having been met, transportation barriers, ADL, IADL, health disorders, Mental Status Questionnaire, psychological symptoms, perceived health
Wan & Odell, 1981, USA [40]	cross-sectional study	1978	1,182	n.a.	55–75: 75% 75+: 25%	60	sex, age, education, retired, economic dependency, ADL, IADL, depression, perceived need for service, transportation barriers, knowledge of service, health insurance coverage
Wolinsky & Coe, 1984, USA [45]	cross-sectional study	1978	1,5899	n.a.	69.9 (mean)	57	sex, age, race, education, retired, labor force, regular source of care, telephone, income, health insurance, region, metropolitan area, limited activity, overall health, BMI
Wolinsky & Johnson, 1991, USA [50]	cross-sectional study	1984	5,151	n.a.	78 (mean)	63.2	age, female, race, telephone, education, health worries and control, healt insurance, residentially stable, population density, social security dependent, perceived health, ADL, body limitations
Wolinsky et al., 1983, USA [51]	cross-sectional study	1980	401	n.a.	74.2 (mean)	66	perceived health, mental orientation, ADL, IADL, sensory functions, nutritional risk, mental health, income, supplemental insurance, preventive care (MD, dentist), locus of control, sex, race, age, index of social position, nutritional knowledge

The methodological and reporting quality of 47.2% of the records was categorized as “high”, 44.4% as “medium” and 8.3% as “low” (Table 2). Apart from criterion two (non-respondents and response rate) and criterion seven (independent assessment of outcome), the majority of the articles met the criteria of methodological and reporting quality (Table 2, S1 Fig, S2 Table). Criterion two (non-respondents and response rate) was met only by five articles [6, 12, 13, 27, 37] and criterion seven (independent assessment of outcome) by eight records [6, 29, 31, 34, 35, 37, 41, 47].

**Table 2:** Results of the quality assessment of the included records (+ = yes, - = no, 0 = unclear).

Author, year	1. repre-sentative-ness of the sample	2. non-respon-dents & response rate	3. sample size	4. ascer-tainment of expo-sure	5. validated or described instrument for exposure	6. compara-bility and confounders	7. indepen-dent assess-ment of outcome	8. follow-up long enough for outcome to occur (only cohort)	9. adequacy of follow up (only cohort)	10. statis-tical test	Global assessment of methodological and reporting quality
Arling, 1985 [8]	+	0	+	+	+	+	-			-	medium
Branch et al., 1981 [42]	+	0	+	+	+	+	-			+	high
Cafferata, 1987 [48]	+	0	+	+	+	+	-			-	medium
Coulton & Frost, 1982 [41]	+	0	+	+	-	+	+	+	-	+	medium
Counte & Glandon, 1991 [28]	0	0	+	+	+	+	-	+	-	+	medium
Crespo-Cebada & Urbanos-Garrido, 2012 [26]	+	0	+	+	+	+	-			+	high
Dalsgaard et al., 2012 [29]	+	n.a.	+	+	+	-	+	+	+	+	high
Eve, 1988 [25]	+	-	+	+	+	+	-	+	-	+	medium
Ezeamama et al., 2015 [46]	+	0	+	+	+	+	-			+	high
Foreman et al., 1998 [9]	-	0	+	+	+	+	-			+	medium
Fritel et al., 2014 [10]	0	-	+	+	+	+	-	+	+	+	medium
Gobbens & van Assen, 2012 [30]	+	-	+	+	+	+	-	+	+	+	high
Goldsteen et al., 1992 [43]	+	-	+	+	+	+	-	+	+	+	high
Hand et al., 2014 [35]	-	0	-	+	+	-	+			-	low
Harris et al., 2004 [34]	+	-	+	+	0	+	+	+	+	+	high
Jordan et al., 2006 [31]	+	0	+	+	+	+	+	+	-	-	medium
Korten & Jacomb, 1998 [32]	0	0	+	+	+	-	-	+	-	-	low
Krause, 1988 [11]	+	0	+	+	+	+	-	+	+	-	medium
Levkoff et al., 1987 [47]	+	-	-	+	+	-	+	+	-	-	medium
Li & Chi, 2011 [12]	+	+	+	+	+	+	-			+	high
Liao et al., 2012 [44]	+	-	+	+	+	+	-	+	+	+	high
Miltiades & Wu, 2008 [49]	-	-	+	+	+	+	-			+	medium
Park, 2012 [38]	0	-	+	+	-	+	-			-	low
Pourat et al., 2000 [39]	0	0	+	+	+	+	-			+	medium
Rennemark et al., 2009 [37]	+	+	+	+	+	+	+			+	high
Ryvicker et al., 2012 [36]	+	-	+	+	+	+	-			+	high
Schafer, 2013 [7]	+	0	+	+	+	+	-			+	high
Schmitz et al., 1997 [6]	-	+	+	+	+	+	+	+	+	-	high
Stoller, 1982 [27]	+	+	+	+	+	+	-			+	high
Strain, 1990 [13]	+	+	+	+	+	+	-			+	high
Suominen-Taipale et al., 2004 [33]	+	-	+	+	+	-	-			+	medium
Wan & Arling, 1983 [24]	0	0	+	+	+	+	-			+	medium
Wan & Odell, 1981 [40]	+	-	+	0	+	+	0			+	medium
Wolinsky & Coe, 1984 [45]	+	0	+	+	+	+	-			+	high
Wolinsky & Johnson, 1991 [50]	+	0	+	+	+	+	-			-	medium
Wolinsky et al., 1983 [51]	+	0	+	+	+	+	-			+	high

### Associations between social relationships and physician utilization (yes vs. no)

Fourty associations between social relationships and the use of physicians were found in thirteen articles (S4 Table). In seven articles, fourteen associations were statistically significant (Table 3). In other words, two thirds of the associations were statistically insignificant.

**Table 3:** Statistically significant associations between social relationship (SR) indicators and physician use (yes/no).

No.	SR dimension	SR category	SR indicator	Author, Year	Statistics	SR coeff. (95%CI, p)
1.	Structural	Marital status—single	Single (0 = married/cohabiting, 1 = single)	Suominen-Taipale et al., 2004 [33]	Odds Ratio	0.6 (0.5–0.8, p<0.05)
2.		Marital status—widowed	Widow (0 = married/cohabiting, 1 = widow)	Suominen-Taipale et al., 2004 [33]	Odds Ratio	0.9 (0.7–1.0, p<0.05)
3.		Marital status—divorced/separated	Divorced/separated (0 = married/cohabiting, 1 = divorced/separated)	Suominen-Taipale et al., 2004 [33]	Odds Ratio	0.7 (0.6–1.0, p<0.05)
4.		Living with others	Living with at least one child (0 = no, 1 = yes)	Li and Chi, 2011 [12]	Odds Ratio	1.38 (1.03–1.84, p<0.05)
5.		Social network size	Social network members (0 = none, 1 = one or more)	Park, 2012 [38]	Odds Ratio	1.28 (n.r., p<0.05)
6.			Household size	Liao et al., 2012 [44]	Random-effect probit model	-0.011 (n.r., p<0.05)
7.		Social cohesion	Neighborhood social cohesion score (range: 5–20)	Ryvicker et al., 2012 [36]	Odds Ratio	1.04 (1.00–1.09, p<0.05)
8.	Functional	Social support (unspecified)	Nonkin supports scale (five items)	Wolinsky and Johnson, 1991 [50]	Unst. OLS coeff.	0.017 (n.r., p<0.05)
9.			Kin supports scale (two items)	Wolinsky and Johnson, 1991 [50]	Unst. OLS coeff.	0.034 (n.r., p<0.05)
10.			Social support scale (0 = strong, 1 = weak)	Fritel et al., 2014 [10]	Odds Ratio	1.4 (1.0–2.0, p<0.05)
11.		Financial support	Receiving financial support (0 = no, 1 = yes)	Li and Chi, 2011 [12]	Odds Ratio	0.47 (0.34–0.65, p<0.001)
12.		Health discussions with others	Discuss health with friends or close relatives (0 = no, 1 = yes)	Fritel et al., 2014 [10]	Odds Ratio	1.5 (1.0–2.1, p<0.05)
13.		Providing financial support	Providing financial support (0 = no, 1 = yes)	Li and Chi, 2011 [12]	Odds Ratio	0.49 (0.33–0.73, p<0.001)
14.		Providing instrumental support	Providing instrumental support (0 = no, 1 = yes)	Li and Chi, 2011 [12]	Odds Ratio	0.73 (0.54–0.99, p<0.01)

SR = social relationship; CI = confidence interval; p = p-value; n.r. = not reported; coeff. = coefficient; Unst. = unstandardized; OLS = ordinary least squares

Seven out of these fourteen associations included variables of the structural dimension of social ties [12, 33, 36, 38, 44]. Suominen-Taipale, Koskinen [33] found consistent and relatively strong negative associations between being single, widowed, divorced or separated and the probability of physician utilization compared to older adults who are married and cohabiting. Li and Chi [12] reported a strong positive association between living with at least one child and the physician use. Regarding the social network size, Park [38] observed a moderate positive association between having social network members and the use of physicians, while Liao, Chang [44] found a weak negative association between the household size and the probability of visiting a physician.

Seven out of fourteen associations included variables of the functional dimension of social relationships [10, 12, 50]. Wolinsky and Johnson [50] found consistently positive, but weak associations between nonkin or kin social support and physician consultations. Fritel, Panjo [10] showed a higher probability of using outpatient care doctors for older people with weak social support. Otherwise, discussing health with friends or close relatives was associated strongly and positively with using health services [10]. Li and Chi [12] analyzed specific forms of social support in their study. For older people receiving or providing financial support or providing instrumental support they observed consistent and strong negative links to the utilization of physicians [12].

### Associations between social relationships and frequency of physician utilization

Ninety-two associations between social relationships and the frequency of physician use were found in twenty-eight articles (S5 Table). In seventeen articles, thirty-seven associations were statistically significant (Table 4). Consequently, more than half of the associations were statistically insignificant.

**Table 4:** Statistically significant associations between social relationship (SR) indicators and frequency of physician visits.

No.	SR dimension	SR category	SR indicator	Author, Year	Statistics	SR coeff. (95%CI, p)
1.	Structural	Marital status—married	Married (0 = not married, 1 = married)	Foreman et al., 1998 [9]	Weighted OLS coeff.	20.454 (n.r., p<0.05)
2.			Married (0 = no, 1 = yes)	Wolinsky and Coe, 1984 [45]	Unst. OLS coeff.	0.091 (n.r., p<0.001)
3.			Married (0 = otherwise, 1 = married)	Miltiades and Wu, 2008 [49]	St. OLS coeff.	0.160 (n.r., p<0.01)
4.		Marital status—widowed	Widowed (0 = no, 1 = yes)	Wolinsky and Coe, 1984 [45]	Unst. OLS coeff.	0.069 (n.r., p<0.01)
5.		Living alone	Alone (0 = otherwise, 1 = lives alone)	Crespo-Cebada and Urbanos-Garrido, 2012 [26]	Count model (elasticity)	0.0149 (n.r., p<0.1)
6.			Lives alone (0 = lives with spouse, 1 = lives alone)	Stoller, 1982 [27]	Unst. OLS coeff.	0.07 (n.r., p<0.1)
7.			Single (0 = cohabiting, 1 = single)	Dalsgaard et al., 2012 [29]	Rates; absolute difference	0.4 (0.2–0.5, p<0.05)
8.			lives alone (0 = no, 1 = yes)	Wolinsky and Coe, 1984 [45]	Unst. OLS coeff.	0.128 (n.r., p<0.001)
9.			Single (0 = cohabiting, 1 = single)	Dalsgaard et al., 2012 [29]	Rates; absolute difference	-0.5 (-0.7–-0.3, p<0.05)
10.			Single (0 = cohabiting, 1 = single)	Dalsgaard et al., 2012 [29]	Rates; absolute difference	0.0 (-0.2–0.2, p<0.05)
11.			Single (0 = cohabiting, 1 = single)	Dalsgaard et al., 2012 [29]	Rates; absolute difference	0.0 (-0.2–0.2, p<0.05)
12.		Living with others	Living with children (0 = not living with children, 1 = living with children)	Foreman et al., 1998 [9]	Weighted OLS coeff.	14.533 (n.r., p<0.05)
13.			living with at least one child (0 = no, 1 = yes)	Li and Chi, 2011 [12]	Count model coeff.	-0.06 (-0.1–-0.01, p<0.01)
14.			lives with others (0 = lives with spouse, 1 = lives with others)	Stoller, 1982 [27]	Unst. OLS coeff.	-0.08 (n.r., p<0.05)
15.			Living arrangement (1 = lives with others except spouse)	Cafferata, 1987 [48]	Unst. OLS coeff.	-0.9 (n.r., p<0.05)
16.		Frequency of social interaction	Telephone contact with friends or relatives (0 = monthly or less, 1 = weekly)	Harris et al., 2004 [34]	Ordered logistic coeff.	1.7 (1.3–2.3, p<0.001)
17.			Telephone contact with friends or relatives (0 = monthly or less, 1 = daily)	Harris et al., 2004 [34]	Ordered logistic coeff.	1.8 (1.4–2.5, p<0.001)
18.			Social network (two items on contact frequency, score range 1–12)	Miltiades and Wu, 2008 [49]	St. OLS coeff.	0.219 (n.r., p<0.01)
19.		Social network size	Social support: network (extent of subject's social network)	Korten and Jacomb, 1998 [32]	St. OLS coeff., Odds Ratio	2.682, 14.6 (2.72–78.39, p<0.05)
20.		Social network (unspecified)	Lubben Social Network Scale: friend (revised)	Pourat et al., 2000 [39]	Exponential Betas	1.11 (n.r., p<0.05)
21.			Lubben Social Network Scale: neighbor (revised)	Pourat et al., 2000 [39]	Exponential Betas	0.93 (n.r., p<0.05)
22.		Social isolation	social isolation (index of social contacts, high score = almost no contact)	Coulton and Frost, 1982 [41]	St. OLS coeff.	-0.6 (n.r., p<0.05)
23.	Functional	Social support (unspecified)	Reliable alliance social provision	Schmitz et al., 1997 [6]	St. OLS coeff.	0.13 (n.r., p<0.05)
24.			Social support (10 forms of assistance)	Arling, 1985 [8]	St. OLS coeff.	0.14 (n.r., p<0.001)
25.		Emotional support	filial piety (1 = not filial—4 = very filial)	Li and Chi, 2011 [12]	Count model coeff.	-0.05 (-0.08–-0.02, p<0.001)
26.		Financial support	receiving financial support (0 = no, 1 = yes)	Li and Chi, 2011 [12]	Count model coeff.	0.05 (0.01–0.10, p<0.01)
27.		Instrumental support	Tangible support	Krause, 1988 [11]	St. OLS coeff.	0.184 (n.r., p<0.01)
28.		Informational support	Informational support	Krause, 1988 [11]	St. OLS coeff.	0.144 (n.r., p<0.05)
29.		Social ties & health discussions	Partner tie—very likely to discuss health (0 = no, 1 = yes)	Schafer, 2013 [7]	Unst. OLS coeff.	1.49 (n.r., p<0.01)
30.			Partner tie—less likely to discuss health (0 = no, 1 = yes)	Schafer, 2013 [7]	Unst. OLS coeff.	1.27 (n.r., p<0.05)
31.			Child ties—very likely to discuss health (0 = no, 1 = yes)	Schafer, 2013 [7]	Unst. OLS coeff.	0.34 (n.r., p<0.05)
32.			Non-kin ties—very likely to discuss health (0 = no, 1 = yes)	Schafer, 2013 [7]	Unst. OLS coeff.	0.37 (n.r., p<0.05)
33.			Non-kin ties—less likely to discuss health (0 = no, 1 = yes)	Schafer, 2013 [7]	Unst. OLS coeff.	0.27 (n.r., p<0.05)
34.		Harmony of social interaction	Relationships with family are harmonious (0 = no, 1 = yes)	Foreman et al., 1998 [9]	Weighted OLS coeff.	-19.538 (n.r., p<0.01)
35.		Respect in social interaction	Receive as much respect from family as deserved (0 = some, little or very little respect, 1 = very much)	Pourat et al., 2000 [39]	Exponential Betas	1.38 (n.r., p<0.05)
36.		Providing instrumental support	providing instrumental support (0 = no, 1 = yes)	Li and Chi, 2011 [12]	Count model coeff.	-0.07 (-0.12–-0.01, p<0.01)
37.		Providing financial support	Providing financial support (0 = no, 1 = yes)	Li and Chi, 2011 [12]	Count model coeff.	0.10 (0.04–0.15, p<0.001)

SR = social relationship; CI = confidence interval; p = p-value; n.r. = not reported; coeff. = coefficient; St. = standardized; Unst. = unstandardized; OLS = ordinary least squares

Twenty-two out of these thirty-seven associations included variables of the structural dimension of social ties. Three studies found positive associations between being married and the frequency of physician visits [9, 45, 49] and one article reported a positive association between being widowed and the frequency of physician consultations [45]. Furthermore, living alone was positively and weakly associated with a higher frequency of using outpatient health services in three records [26, 27, 45]. Dalsgaard, Vedsted [29] found no, positive and negative differences for older adults who are living alone depending on their age and gender. Living with others (e.g., child or others except spouse) was associated negatively with the frequency of utilizing physicians in three studies [12, 27, 48]. Foreman, Yu [9] reported a strong and positive association between living with children and the frequency of health services use. The size of the social network was positively and strongly associated with a higher frequency of physician visits [32]. Counting friends or neighbors amongst their social networks, older adults reported a higher number of physician consultations [39]. Coulton and Frost [41] found out that socially isolated older people showed a lower number of physician contacts than socially integrated older adults. Moreover, Harris, Cook [34] and Militades and Wu [49] observed positive associations between higher contact frequencies in social networks and the frequency of physician use.

Fifteen out of thirty-seven associations included variables of the functional dimension of social relationships. Two studies showed positive and weak associations between received social support and the frequency of physician utilization [6, 8]. Financial, instrumental or informational support was associated weakly with more physician visits [11, 12]. Emotional support was associated with less consultations [12]. Schafer [7] reported moderate to strong and positive associations between the likelihood of discussing health and the frequency of physician use taking several social ties into consideration (partner, children, non-kin). Harmonious social relationships decreased the frequency of physician visits [9] and respectful social ties increased the use rate [39].

Li and Chi [12] investigated the association between providing social support and the frequency of using physicians. Providing instrumental support was associated negatively and weakly. The provision of financial support was linked positively and weakly.

## Discussion

### Summary of findings

This review provides a comprehensive overview and furthers the understanding of the association between social relationships and health services use among older adults (50 years and older). The first objective of this study was to systematically review social relationships associated with the utilization of outpatient care services of older people. The second aim was to evaluate magnitude and consistency of the associations between social ties and health services use.

We included thirty-six records on thirty-five different studies reporting structural and functional dimensions of social relationships linked to the utilization of health services into our analyses. In most cases empirical evidence was insufficient and for several of the social tie variables inconsistent results were found. Taking into account the fully adjusted model, associations between use measures and social relationship variables were for the most part weak and statistically insignificant. Potentially, associations were underestimated by that strict criterion.

Overall, most of the studies focused on associations between social ties and frequency of physician use. The structural dimension of social relationships and its association with physician visits (use and frequency of use) was investigated far more often than the functional dimension. Though a substantial number of social relationship dimensions were explored until now, none of the included studies included a holistic approach of social tie measures (degree of integration, received and perceived social support) [15] and theirs links to health services utilization.

The majority of the associations between structural dimensions of social relationships and the use of physicians were positive and moderate in strength. The associations between functional dimensions of social relationships and the probability of using physician services were inconsistent and varied in strength. For the most part, social relationship variables assigned to the structural dimension were positively and weakly to moderately associated with the frequency of physician visits. Functional aspects of social relationships also tended to have positive associations with the frequency of physician utilization. The associations were weak or moderate in strength. All in all, the current status of quantitative data was insufficient to draw precise and generalizable conclusions.

Our review reveals that the link between various social relationship indicators and health care use as well as frequency of use have been investigated in few studies. This clearly indicates that further research is needed.

### Limitations

Including a broad range of seven medical and sociological databases, we were able to minimize the risk of missing relevant articles. Nevertheless, the risk of publication bias is still existent. More than half of the studies (64%) were performed in North America, and therefore, findings cannot be generalized. Since the majority of included studies (61%) had cross-sectional design, conclusions concerning causal relations are not possible.

Due to the fact that ten studies did not (four studies) or did not clearly meet (six studies) the quality criterion of representativeness and thirty articles did not (twelve studies) or did not clearly report (eighteen studies) information on non-respondents and response rate, the results were moderately robust. Overall, the methodological and reporting quality of the studies was mostly categorized as medium or high (92%).

Most of the studies referred to one year of physician use. Still, the range of the utilization variable was substantial between the studies (from 15 days to two years). As the time span was quite long in some studies, and considering the older age of the interviewed individuals, risk of memory bias was existent, particularly, if the information on consultations was not compared to medical records (twenty-seven studies).

Since there were no consistent measures of predictors (social relationships) and outcome variables (use and frequency of outpatient care visits), data was analyzed systematically, but descriptively. A prerequisite of meta-analyses is a high level of accordance across the included studies regarding independent and dependent variable measures and data analysis approaches [52]. Due to the heterogeneity of the included studies (e.g. study designs, sampling procedures, data collection methods, definition of outcome and exposure variables, confounders, quality of studies, statistical analysis and reporting) a meta-analysis was not conducted. In most cases the associations were small and statistically not significant. The current status of evidence is insufficient and partly inconsistent.

Unfortunately, analyses of group-differences concerning age, gender, and chronic conditions could not conducted on the basis of the review material.

## Conclusions

Social relationships can increase or decrease the probability to consult a physician, and they can influence the frequency of visits. All in all, older people who are structurally integrated by social relationships are more likely to consult a physician at all and to contact a physician more often. Functional aspects of social relationships, depending on the form of social support, can increase or decrease the probability of physician use. Older adults who are experiencing social support tend to have a higher rate of physician visits than older people without any or less support.

On the one hand, this could be read as good news, since structural and functional aspects of social relationships tend to enable the utilization of health services, and thereby potentially foster older adults’ health. Social relationships could offer informational, instrumental and emotional resources with regard to health, health care services and treatments. On the other hand, considering increasing numbers of single-person-households and an increasing risk of loneliness and social isolation in older age [53], this could be interpreted as a cause of concern, since older individuals who are not socially integrated may not find their way to health care services. The results do not include information about the adequacy of health care regarding access to health services, extent of health treatment, and quality of health care.

Social ties have an impact on the patient’s motives for a consultation and on the patient’s compliance regarding future visits for treatment, prevention or rehabilitation [54, 55]. Consequently, health care practitioners should consider information on patient’s social environments into their clinical routine. By default, physicians should assess social networks among the elderly screening for social resources or social needs of support. Furthermore, relevant stakeholders (e.g., physicians, public health institutions and health insurance companies) need to find ways to ensure that older adults can use outpatient care services regardless of their structural and functional level of social integration.

The variety of dimensions of social relationships presented in this review illustrates that utilization of outpatient health care services is a complex social process. Besides methodological challenges, the complex picture of social tie’s impact on health care utilization bases on the fact that relationships are not always of positive virtue [56, 57]. In contrary, “some of the most powerful impacts on health [and health services use] that social relationships may have, are through acts of abuse, violence, and trauma” [14]. This fact may represent a possible explanation for the inconsistent pattern of social relationships on health services use among older adults.

Furthermore, the inconclusive results demonstrate that measuring social relationships and their influence on health services use is a challenging methodological endeavor. Future health services research should accentuate social relationship variables more in detail, and not only in terms of structure and quantity, but also according to functional and quality aspects of social relationships.

The relatively low number of included studies indicates a deficit of elaborated observational studies dealing with the role of social relationships for the utilization of health services among older populations. The majority of the identified studies have a cross-sectional design investigating a number of possible social relationships of health services use. It is crucial to determine social ties for health services use more clearly and to identify causal relations, especially in the form of prospective cohort studies.

Methodologically, it can be constructive to directly connect the question of social relationships and health care utilization to the scientific debate of health care inequalities [58–62] by conducting mediator or moderator analyses to create further clarity. This may complement the identification and understanding of social inequalities in health services utilization. In the future, this can be directed into new approaches to reduce social inequalities in health services utilization and to offer needs-based access to health care and adequate levels of treatment.

## Supporting information

S1 File Protocol PROSPERO. (PDF)

S1 Text Search syntax for PubMed. (DOCX)

S1 Fig Methodological and reporting quality of included records. (DOCX)

S1 Table PRISMA checklist. (DOC)

S2 Table Checklist quality assessment. (DOCX)

S3 Table Included and excluded full-text studies. (XLSX)

S4 Table Associations with physician use. (XLSX)

S5 Table Associations with frequency of physician use. (XLSX)

## References

[1] Beaglehole R, Irwin A, Prentice T. The World Health Report 2003: Shaping the Future. Geneva: World Health Organization, 2003.

[2] Freid VM, Bernstein AB. Health care utilization among adults aged 55–64 years: How has it changed over the past 10 years? Hyattsville: National Center for Health Statistics, 2010 32. 20356438

[3] Andersen RM. Revisiting the Behavioral Model and Access to Medical Care: Does it Matter? J Health Soc Behav. 1995;36(1):1–10. 7738325

[4] Abbott P, Sapsford R. Living on the Margins: Older People, Place and Social Exclusion. Policy Studies. 2005;26(1):29–46.

[5] Pescosolido BA. Beyond rational choice: the social dynamics of how people seek help. Am J Sociol. 1992;97(4):1096–138.

[6] Schmitz MF, Russell DW, Cutrona CE. Perceived Social Support and Social Network Influences on Physician Utilization Among the Elderly. Research in the Sociology of Health Care. 1997;14:249–72.

[7] Schafer MH. Discussion networks, physician visits, and non-conventional medicine: probing the relational correlates of health care utilization. Soc Sci Med. 2013;87:176–84. doi: 10.1016/j.socscimed.2013.03.031 23631793 10.1016/j.socscimed.2013.03.031

[8] Arling G. Interaction effects in a multivariate model of physician visits by older people. Med Care. 1985;23(4):361–71. 3872978 10.1097/00005650-198504000-00008

[9] Foreman SE, Yu LC, Barley D, Chen LW. Use of health services by Chinese elderly in Beijing. Med Care. 1998;36(8):1265–82. 9708598 10.1097/00005650-199808000-00014

[10] Fritel X, Panjo H, Varnoux N, Ringa V. The individual determinants of care-seeking among middle-aged women reporting urinary incontinence: analysis of a 2273-woman cohort. Neurourol Urodynam. 2014;33(7):1116–22. 10.1002/nau.22461 PMC5225260 23818427

[11] Krause N. Stressful life events and physician utilization. J Gerontol. 1988;43(2):S53–61. 3346529 10.1093/geronj/43.2.s53

[12] Li Y, Chi I. Correlates of physician visits among older adults in China: the effects of family support. J Aging Health. 2011;23(6):933–53. doi: 10.1177/0898264311401390 21617127 10.1177/0898264311401390

[13] Strain LA. Physician visits by the elderly: Testing the Anderson-Newman framework. Can J Sociol. 1990;15(1):19.

[14] Berkman LF, Glass T, Brissette I, Seeman TE. From social integration to health: Durkheim in the new millennium. Soc Sci Med. 2000;51(6):843–57. 10972429 10.1016/s0277-9536(00)00065-4

[15] Holt-Lunstad J, Smith TB, Layton JB. Social relationships and mortality risk: a meta-analytic review. PLoS Med. 2010;7(7):e1000316 doi: 10.1371/journal.pmed.1000316 20668659 10.1371/journal.pmed.1000316 PMC2910600

[16] Blazer DG. Social support and mortality in an elderly community population. Am J Epidemiol. 1982;115(5):684–94. 7081200 10.1093/oxfordjournals.aje.a113351

[17] Hemingway H, Marmot M. Evidence based cardiology: psychosocial factors in the aetiology and prognosis of coronary heart disease. Systematic review of prospective cohort studies. Bmj. 1999;318(7196):1460–7. 10346775 10.1136/bmj.318.7196.1460 PMC1115843

[18] House JS, Landis KR, Umberson D. Social relationships and health. Science. 1988;241(4865):540–5. 3399889 10.1126/science.3399889

[19] Moher D, Liberati A, Tetzlaff J, Altman DG. Preferred reporting items for systematic reviews and meta-analyses: the PRISMA statement. PLoS Med. 2009;6(7):e1000097 doi: 10.1371/journal.pmed.1000097 19621072 10.1371/journal.pmed.1000097 PMC2707599

[20] Wells GA, Shea B, O'Connell D, Peterson J, Welch V, Losos M, et al. The Newcastle-Ottawa Scale (NOS) for assessing the quality of nonrandomised studies in meta-analyses 2000 [cited 2016 July 30th]. http://www.ohri.ca/programs/clinical_epidemiology/oxford.asp.

[21] Herzog R, Alvarez-Pasquin MJ, Diaz C, Del Barrio JL, Estrada JM, Gil A. Are healthcare workers' intentions to vaccinate related to their knowledge, beliefs and attitudes? A systematic review. BMC Public Health. 2013;13:154 doi: 10.1186/1471-2458-13-154 23421987 10.1186/1471-2458-13-154 PMC3602084

[22] Jüni P, Altman DG, Egger M. Assessing the quality of randomized controlled trials In: Egger M, Smith GD, Altman DG, editors. Systematic reviews in Health Care Meta-Analysis in Context. London: BMJ Publishing Group; 2003 p. 87–108.

[23] Murad MH, Mustafa RA, Schünemann HJ, Sultan S, Santesso N. Rating the certainty in evidence in the absence of a single estimate of effect. Evid Based Med. 2017:ebmed-2017-110668. 10.1136/ebmed-2017-110668 PMC5502230 28320705

[24] Wan TTH, Arling G. Differential Use of Health Services among Disabled Elderly. Res Aging. 1983;5(3):411–31.

[25] Eve SB. A Longitudinal-Study of Use of Health-Care Services among Older Women. J Gerontol. 1988;43(2):M31–M9. 2964467 10.1093/geronj/43.2.m31

[26] Crespo-Cebada E, Urbanos-Garrido RM. Equity and equality in the use of GP services for elderly people: the Spanish case. Health Policy. 2012;104(2):193–9. doi: 10.1016/j.healthpol.2011.10.007 22071455 10.1016/j.healthpol.2011.10.007

[27] Stoller EP. Patterns of physician utilization by the elderly: a multivariate analysis. Med Care. 1982;20(11):1080–9. 7144271 10.1097/00005650-198211000-00003

[28] Counte MA, Glandon GL. A panel study of life stress, social support, and the health services utilization of older persons. Med Care. 1991;29(4):348–61. 2020203 10.1097/00005650-199104000-00004

[29] Dalsgaard EM, Vedsted P, Fenger-Gron M, Sandbaek A, Vestergaard M. Socioeconomic position and contact to general practice among persons with diabetes. Prim Care Diabetes. 2012;6(4):313–8. doi: 10.1016/j.pcd.2012.06.002 22809561 10.1016/j.pcd.2012.06.002

[30] Gobbens RJJ, van Assen MALM. Frailty and its prediction of disability and health care utilization: The added value of interviews and physical measures following a self-report questionnaire. Arch Gerontol Geriat. 2012;55(2):369–79. 10.1016/j.archger.2012.04.008 22595766

[31] Jordan K, Jinks C, Croft P. A prospective study of the consulting behaviour of older people with knee pain. Br J Gen Pract. 2006;56(525):269–76. 16611515 PMC1832234

[32] Korten AE, Jacomb PA. Predictors of GP service use: a community survey of an elderly Australian sample. Australian N Z J Public Health. 1998;22(5):609. 9744218 10.1111/j.1467-842x.1998.tb01447.x

[33] Suominen-Taipale AL, Koskinen S, Martelin T, Holmen J, Johnsen R. Differences in older adults' use of primary and specialist care services in two Nordic countries. Eur J Public Health. 2004;14(4):375–80. doi: 10.1093/eurpub/14.4.375 15542872 10.1093/eurpub/14.4.375

[34] Harris T, Cook DG, Victor CR, Beighton C, Dewilde S, Carey IM. Linking survey data with computerised records to predict consulting by older people. Br J Gen Pract. 2004;54(509):928–31. 15588539 PMC1326112

[35] Hand C, McColl MA, Birtwhistle R, Kotecha JA, Batchelor D, Barber KH. Social isolation in older adults who are frequent users of primary care services. Can Fam Physician. 2014;60(6):e322, e4–9. 24925967 PMC4055344

[36] Ryvicker M, Gallo WT, Fahs MC. Environmental factors associated with primary care access among urban older adults. Soc Sci Med. 2012;75(6):914–21. 22682664 10.1016/j.socscimed.2012.04.029 PMC3383917

[37] Rennemark M, Holst G, Fagerstrom C, Halling A. Factors related to frequent usage of the primary healthcare services in old age: findings from The Swedish National Study on Aging and Care. Health Soc Care Community. 2009;17(3). 10.1111/j.1365-2524.2008.00829.x 19207603

[38] Park JM. Equity of access to primary care among older adults in Incheon, South Korea. Asia-Pac J Public Health. 2012;24(6):953–60. doi: 10.1177/1010539511409392 21653609 10.1177/1010539511409392

[39] Pourat N, Lubben J, Yu H, Wallace S. Perceptions of health and use of ambulatory care: differences between Korean and White elderly. J Aging Health. 2000;12(1):112–34. doi: 10.1177/089826430001200106 10848128 10.1177/089826430001200106

[40] Wan TTH, Odell BG. Factors Affecting the Use of Social and Health Services Among the Elderly. Ageing Soc. 1983;1(1):95–115.

[41] Coulton C, Frost AK. Use of Social and Health-Services by the Elderly. J Health Soc Behav. 1982;23(4):330–9. 7161472

[42] Branch L, Jette A, Evashwick C, Polansky M, Rowe G, Diehr P. Toward understanding elders' health service utilization. J Community Health. 1981;7(2):80–92. 7328199 10.1007/BF01323227

[43] Goldsteen R, Counte MA, Glandon GL, Goldsteen K. Desirable Life Events and Physician Utilization among Older American Men and Women. J Aging Stud. 1992;6(2):149–63.

[44] Liao P-A, Chang H-H, Yang F-A. Does the Universal Health Insurance Program Affect Urban-Rural Differences in Health Service Utilization Among the Elderly? Evidence From a Longitudinal Study in Taiwan. J Rural Health. 2012;28(1):84–91. doi: 10.1111/j.1748-0361.2011.00363.x 22236318 10.1111/j.1748-0361.2011.00363.x

[45] Wolinsky FD, Coe RM. Physician and Hospital Utilization among Noninstitutionalized Elderly Adults—an Analysis of the Health Interview Survey. J Gerontol. 1984;39(3):334–41. 6715812 10.1093/geronj/39.3.334

[46] Ezeamama AE, Elkins J, Simpson C, Smith SL, Allegra JC, Miles TP. Indicators of resilience and healthcare outcomes: findings from the 2010 health and retirement survey. Qual Life Res. 2016;25(4):1007–15. doi: 10.1007/s11136-015-1144-y 26475139 10.1007/s11136-015-1144-y

[47] Levkoff SE, Cleary PD, Wetle T. Differences in Determinants of Physician Use between Aged and Middle-Aged Persons. Med Care. 1987;25(12):1148–60. 3501048 10.1097/00005650-198712000-00004

[48] Cafferata GL. Marital-Status, Living Arrangements, and the Use of Health-Services by Elderly Persons. J Gerontol. 1987;42(6):613–8. 3680880 10.1093/geronj/42.6.613

[49] Miltiades HB, Wu B. Factors affecting physician visits in Chinese and Chinese immigrant samples. Soc Sci Med (1982). 2008;66(3):704–14. 10.1016/j.socscimed.2007.10.016 17996348

[50] Wolinsky FD, Johnson RJ. The Use of Health-Services by Older Adults. J Gerontol. 1991;46(6):S345–S57. 1940101 10.1093/geronj/46.6.s345

[51] Wolinsky FD, Coe RM, Miller DK, Prendergast JM, Creel MJ, Chavez MN. Health-Services Utilization among the Noninstitutionalized Elderly. J Health Soc Behav. 1983;24(4):325–37. 6668412

[52] Hedges LV, Olkin I. Vote-counting methods in research synthesis. Psychol Bull. 1980;88(2):359.

[53] Laporte A, Nauenberg E, Shen L. Aging, social capital, and health care utilization in Canada. Health EconPolicy Law. 2008;3(Pt 4):393–411. 10.1017/S1744133108004568 18793479

[54] Cornwell EY, Waite LJ. Social network resources and management of hypertension. J Health Soc Behav. 2012;53(2):215–31. doi: 10.1177/0022146512446832 22660826 10.1177/0022146512446832 PMC3727627

[55] Zhang AY, Siminoff LA. The role of the family in treatment decision making by patients with cancer. Oncol Nurs Forum. 2003;30(6):1022–8. doi: 10.1188/03.ONF.1022-1028 14603359 10.1188/03.ONF.1022-1028

[56] Rook K. Stressful aspects of older adults' social relationships: current theory and research In: Stephens AP, Crowther JH, Hobfoll SE, Tennenbaum DL, editors. Stress and coping in later-life families. New York: Hemisphere; 1990 p. 173–92.

[57] von dem Knesebeck O, Dragano N, Moebus S, Jockel KH, Erbel R, Siegrist J. [Stressful experiences in social relationships and ill health]. Psychother Psych Med. 2009;59(5):186–93. 10.1055/s-2008-1067421 18600613

[58] Aida J, Kondo K, Kondo N, Watt RG, Sheiham A, Tsakos G. Income inequality, social capital and self-rated health and dental status in older Japanese. Soc Sci Med. 2011;73(10):1561–8. doi: 10.1016/j.socscimed.2011.09.005 21982631 10.1016/j.socscimed.2011.09.005

[59] Dahl E, Malmberg-Heimonen I. Social inequality and health: the role of social capital. Sociol Health Illn. 2010;32(7):1102–19. doi: 10.1111/j.1467-9566.2010.01270.x 21044094 10.1111/j.1467-9566.2010.01270.x

[60] Fone D, Dunstan F, Lloyd K, Williams G, Watkins J, Palmer S. Does social cohesion modify the association between area income deprivation and mental health? A multilevel analysis. Int J Epidemiol. 2007;36(2):338–45. doi: 10.1093/ije/dym004 17329315 10.1093/ije/dym004

[61] von dem Knesebeck O, Geyer S. Emotional support, education and self-rated health in 22 European countries. BMC Public Health. 2007;7:272 doi: 10.1186/1471-2458-7-272 17908313 10.1186/1471-2458-7-272 PMC2048950

[62] Melchior M, Berkman LF, Niedhammer I, Chea M, Goldberg M. Social relations and self-reported health: a prospective analysis of the French Gazel cohort. Soc Sci Med. 2003;56(8):1817–30. 12639598 10.1016/s0277-9536(02)00181-8

