# Supplementary material for: Social relationships and physician utilization among older adults—A systematic review
Source: PLoS One. 2017 Sep 28;12(9):e0185672. doi: 10.1371/journal.pone.0185672 (PMC5619811; doi:10.1371/journal.pone.0185672)
Supplement: S1 File — (PDF) [file pone.0185672.s001.pdf]

Social relationships and physician utilization among older adults: a systematic review

Daniel Bremer, Laura Inhestern, Olaf von dem Knesebeck

Citation

Daniel Bremer, Laura Inhestern, Olaf von dem Knesebeck. Social relationships and physician utilization among older adults: a systematic review. PROSPERO 2016:CRD42016036004 Available from
http://www.crd.york.ac.uk/PROSPERO_REBRANDING/display_record.asp?ID=CRD42016036004

Review question(s)

The objective of this review is to assess associations between social relationships and outpatient care utilization among older adults.

The first aim of this systematic review is to provide an overview of studies dealing with outpatient care utilization among older adults associated with various dimensions of social relationships.

The second aim is to evaluate the sufficiency and consistency of associations between social ties and health services use.

Searches

The following databases were searched:

- PubMed

- PsycINFO

- CINAHL

- IBSS

- SocINDEX

- Sociological Abstracts

- ASSIA

The search strategies were adjusted to each database without any restrictions to the date of publication. The searches are limited to English and German, journal articles and humans. References of relevant articles will be searched for further studies.

Types of study to be included

Observational studies:

- Cross-sectional studies

- Case-control studies

- Cohort studies (retrospective and prospective)

Condition or domain being studied

The review focuses on social relationships associated with outpatient health services among older populations (50 years and older).

Participants/ population
The review will include observational studies which include a community-dwelling population with the age of fifty and older reporting outpatient visits in a certain period of time while characterizing social relationships of the participants (e.g., quantity and/ or quality of social contacts). The population will not be restricted to specific diseases.

Intervention(s), exposure(s)
Not applicable

Comparator(s)/ control
Not specified

Outcome(s)
Primary outcomes
outpatient visits (number of visits, visits vs. none)

Secondary outcomes
none

Data extraction, (selection and coding)
Two researchers extract data independently from included full texts on the basis of a data extraction form.

The extraction form will include the following variables:

author, year, country, research design, study year (follow-up if applicable), sample size, response rate, age, gender, outcome, social relationship variables, and confounders in the fully adjusted model.

Risk of bias (quality) assessment
Two authors will perform independently the assessment of quality of the included studies using a global assessment of the methodological quality.

The quality assessment, including the methodological and reporting quality, is based on a checklist following the Newcastle-Ottawa-Scale and its adaptation of Herzog et al. (2013). The checklist includes the three sections “selection”, “comparability and confounders” and “outcome”.

We will solve disagreements about the quality by discussion and if necessary with the help of a third researcher.

Strategy for data synthesis
Following Deeks et al. (2009) a narrative synthesis will be conducted by performing vote counting regarding social characteristics (positive, negative and neutral findings). Nevertheless, data will be controlled for performing a meta-analysis. We expect substantial variations in study designs, sampling procedures, data collection methods, outcome variables and social characteristics.

Analysis of subgroups or subsets
If possible, we will determine stratifications on gender, age and chronic disease.

Contact details for further information
Daniel Bremer

Martinistr. 52, 20246 Hamburg

da.bremer@uke.de

Organisational affiliation of the review
Center for Health Care Research, University Medical Center Hamburg-Eppendorf

http://www.uke.de/english/research/key-research-areas/center-for-health-care-research-chcr/index.html

Review team

Mr Daniel Bremer, Center for Health Care Research & Department of Medical Psychology, University Medical Center Hamburg-Eppendorf
Miss Laura Inhestern, Department of Medical Psychology, University Medical Center Hamburg-Eppendorf
Professor Olaf von dem Knesebeck, Department of Medical Sociology, University Medical Center Hamburg-Eppendorf

Anticipated or actual start date

27 January 2016

Anticipated completion date

27 October 2016

Funding sources/sponsors

none

Conflicts of interest

None known

Language

English

Country

Germany

Subject index terms status

Subject indexing assigned by CRD

Subject index terms

Aged; Drive; Humans; Work

Stage of review

Completed but not published

Date of registration in PROSPERO

04 March 2016

Date of publication of this revision

28 October 2016

Stage of review at time of this submission	Started	Completed
Preliminary searches	Yes	Yes
Piloting of the study selection process	Yes	Yes
Formal screening of search results against eligibility criteria	Yes	Yes
Data extraction	Yes	Yes
Risk of bias (quality) assessment	Yes	Yes
Data analysis	Yes	Yes

PROSPERO
International prospective register of systematic reviews
The information in this record has been provided by the named contact for this review. CRD has accepted this information in good faith and registered the review in PROSPERO. CRD bears no responsibility or liability for the content of this registration record, any associated files or external websites.
