# Supplementary material for: Social relationships and physician utilization among older adults—A systematic review
Source: PLoS One. 2017 Sep 28;12(9):e0185672. doi: 10.1371/journal.pone.0185672 (PMC5619811; doi:10.1371/journal.pone.0185672)
Supplement: S1 Text — (DOCX) [file pone.0185672.s002.docx]

**Search syntax on “Title” and “Abstract” for PubMed**

(social relation* OR social support OR social network* OR social capital OR social integration OR social contact* OR social tie* OR family network* OR family support OR network analysis OR support network OR social inequalit* OR social disparit*) **AND** (visit* OR consultation* OR help seek* OR usage OR utilisation OR utilization OR uptake OR “health care use” OR “health service use” OR “health services use” OR “utilization” OR “health services needs and demand”) **AND** (primary care* OR outpatient care* OR ambulatory care* OR general practi* OR family practi* OR family doctor* OR family physician* OR physician* OR geriatric* OR internal medicine OR general medicine OR family medicine) **AND** (old* OR elder* OR aged OR ageing OR aging OR oldest old OR community-dwelling) **AND** (english OR german)
