# Supplementary material for: Social relationships and physician utilization among older adults—A systematic review
Source: PLoS One. 2017 Sep 28;12(9):e0185672. doi: 10.1371/journal.pone.0185672 (PMC5619811; doi:10.1371/journal.pone.0185672)
Supplement: S1 Fig — (DOCX) [file pone.0185672.s003.docx]

**Methodological and reporting quality of included records**
