# Supplementary material for: Social relationships and physician utilization among older adults—A systematic review
Source: PLoS One. 2017 Sep 28;12(9):e0185672. doi: 10.1371/journal.pone.0185672 (PMC5619811; doi:10.1371/journal.pone.0185672)
Supplement: S2 Table — (DOCX) [file pone.0185672.s005.docx]

**Checklist quality assessment**

| Assessment of the methodological and reporting quality of studies |  |  |  |
| --- | --- | --- | --- |
| SELECTION |  |  |  |
| 1. Is the representativeness of the sample/ exposed cohort truly or somewhat representative of the average in the target population? | yes | no | unclear |
| 2. Is the comparability between respondents and non-respondents characteristics established, and is the response rate is satisfactory? | yes | no | unclear |
| 3. Is the sample size justified and satisfactory? | yes | no | unclear |
| 4. Is the ascertainment of exposure based on secure records, structured or written self-report? | yes | no | unclear |
| 5. Is the instrument for exposure validated or described? | yes | no | unclear |
| COMPARABILITY & CONFOUNDERS | yes | no | unclear |
| 6. Does the study control for more than one confounding factor, and are they comparable in different groups/ cohorts? | yes | no | unclear |
| OUTCOME | yes | no | unclear |
| 7. Is the assessment of outcome based on or supported by registered medical utilization data? | yes | no | unclear |
| 8. Is the follow-up long enough for outcome to occur? (ONLY COHORT) | yes | no | unclear |
| 9. Is the follow-up of cohorts adequate (attrition bias)? (ONLY COHORT) | yes | no | unclear |
| 10. Is the statistical test used to analyze the data clearly described and appropriate, and is the measurement of the association presented? | yes | no | unclear |
